# Combined Association of Time to Treatment, Guideline Concordance, and Neighborhood Vulnerability with Localized Colon Cancer, Non-small-cell Lung Cancer, and Pancreatic Cancer Survival

**DOI:** 10.1245/s10434-026-19760-5

**Published:** 2026-05-17

**Authors:** Muhammad Sohaib Khan, Tammy Leonard, Natalie Williams, Saad Mallick, Jennie Meier, Gilbert Z. Murimwa, Sarah Zamarripa, Herbert J. Zeh, Patricio M. Polanco

**Affiliations:** 1 Division of Surgical Oncology, Department of Surgery, UT Southwestern Medical Center, Dallas, TX USA; 2 Peter O’Donnell Jr. School of Public Health, UT Southwestern Medical Center, Dallas, TX USA; 3 Moncrief Cancer Institute, UT Southwestern Medical Center, Dallas, TX USA

**Keywords:** Guideline concordance, Social vulnerability, Time to treatment, Survival

## Abstract

**Background:**

Patients with social barriers have poor cancer outcomes. The combined relationship of cancer survival with guideline concordance, time to treatment, and social vulnerability has not been studied. Our objective was to determine whether optimal care is associated with survival and whether this relationship differs with social vulnerability.

**Methods:**

Adult patients diagnosed with localized colon cancer, non-small-cell lung cancer (NSCLC), or pancreatic cancer from 2006 to 2016 and followed until 2021 were identified from California and Texas Cancer Registries and analyzed with the census-tract level Social Vulnerability Index. Optimal care (guideline-concordant treatment initiated within 60 days of diagnosis) was used to create an interaction variable by combining it with the Social Vulnerability Index. A multivariable Cox proportional hazards model was used for survival analysis.

**Results:**

Of the 100,294 patients, 68.7% of those with colon cancer, 22.6% of those with pancreatic cancer, and 70.6% of those with NSCLC received optimal care. Cox models showed that patients who received optimal care had the lowest hazard ratios (HRs) for death compared with those who received least optimal care: colon cancer, HR 0.45 (95% confidence interval [CI] 0.36–0.57), NSCLC, HR 0.35 (95% CI 0.32–0.38), pancreatic cancer, HR 0.46 (95% CI 0.39–0.54). On interaction analysis, for patients with colon cancer or NSCLC who received optimal care, the risk of death was higher for patients who lived in the most vulnerable neighborhoods than for those who did not.

**Conclusion:**

Care that is both guideline concordant and timely is associated with the highest overall survival for localized cancer. However, this association is reduced for patients with colon cancer or NSCLC residing in the most vulnerable neighborhoods.

**Supplementary Information:**

The online version contains supplementary material available at 10.1245/s10434-026-19760-5.

Cancer is the second leading cause of death in the United States and responsible for over 600,000 deaths annually.^1^ These deaths are unevenly distributed, with certain sub-populations reported to have disproportionately higher cancer-related death rates.^1^ Although recent advances in therapeutics have significantly improved survival expectations, the ongoing lack of equitable access to optimal cancer care continues to diminish survival benefits and exacerbate disparities in disadvantaged populations. Therefore, to improve cancer survival, population-based studies to identify factors that limit vulnerable patients from receiving optimal cancer care are invaluable.

Survival after cancer diagnosis is a function of multiple factors that can be classified as either upstream or downstream.^2^ Cancer care or treatment is a key downstream factor affecting survival. However, the delivery of optimal cancer care has faced significant challenges, warranting its recognition as one of the goals of the National Cancer Plan.^3^ Numerous disparities related to the delivery of guideline-concordant care and delays in treatment initiation have been identified.^4–6^ Understanding the relationship of these attributes together is important as it has significant implications, especially for socially vulnerable patients. In addition, cancer inequities are believed to originate in more upstream factors.^7^ Socioeconomic status, household characteristics, race and ethnicity, housing, and transportation combine to create social circumstances in which patients with cancer live and receive treatment.^7^ Although recent studies have investigated upstream and downstream factors separately, their joint association has not been studied.

Our study examined the combined relationship of timeliness and guideline concordance of treatment and neighborhood vulnerability with cancer survival. We hypothesized that optimal cancer care, defined as timely and guideline concordant, is positively associated with survival. Prior research has only analyzed these aspects separately. We also hypothesized that the positive relationship of optimal cancer care may be reduced for patients who live in vulnerable neighborhoods. For this, we examined heterogeneity in the association of optimal care across neighborhoods with varied levels of social vulnerability to provide evidence about the enduring relationship of upstream social vulnerabilities.

## Methods

### Setting, Data Source, and Study Population

The study was performed after receiving approval from the University of Texas Southwestern institutional review board and followed the Strengthening the Reporting of Observational Studies in Epidemiology (STROBE) guidelines.^8^ This was a retrospective cohort study that included the top three leading causes of cancer deaths for men and women combined: colon cancer, non-small-cell lung cancer (NSCLC), and pancreatic cancer. Patient-level outcomes and covariates were obtained from the California and Texas Cancer Registries, two of the largest cancer registries that are Gold Certified by the North American Association of Central Cancer Registries.^9^ Both states have laws that mandate the reporting of all cancer cases diagnosed in the state. For patients diagnosed between 2006 and 2016, all patients were observed until 2021 or death if they died before 2021. This timeframe was selected to ensure that each patient had a minimum of 5 years of actual survival data, as both registries ascertain death through linkage with state vital statistics and the National Death Index.

Our neighborhood-level exposure measure was the Social Vulnerability Index (SVI), developed by the Centers for Disease Control and Prevention. The SVI is a comprehensive metric of social vulnerability that uses data from 5-year estimates of the American Community Survey. It is based on 16 characteristics grouped into four themes: socioeconomic status, household characteristics, racial and ethnic minority status, and housing type and transportation.^10^ Census tracts are assigned a percentile rank based on their relative performance across all themes, with higher percentiles corresponding with more vulnerability. Federal Information Processing Standards codes were used to link SVI data to cancer registry data. To maintain congruency with years, 2010 SVI data were linked with cancers diagnosed from the years 2006 to 2010, whereas 2016 SVI data were linked for the years 2011 to 2016.

### Patient Selection

**Fig. 1:**
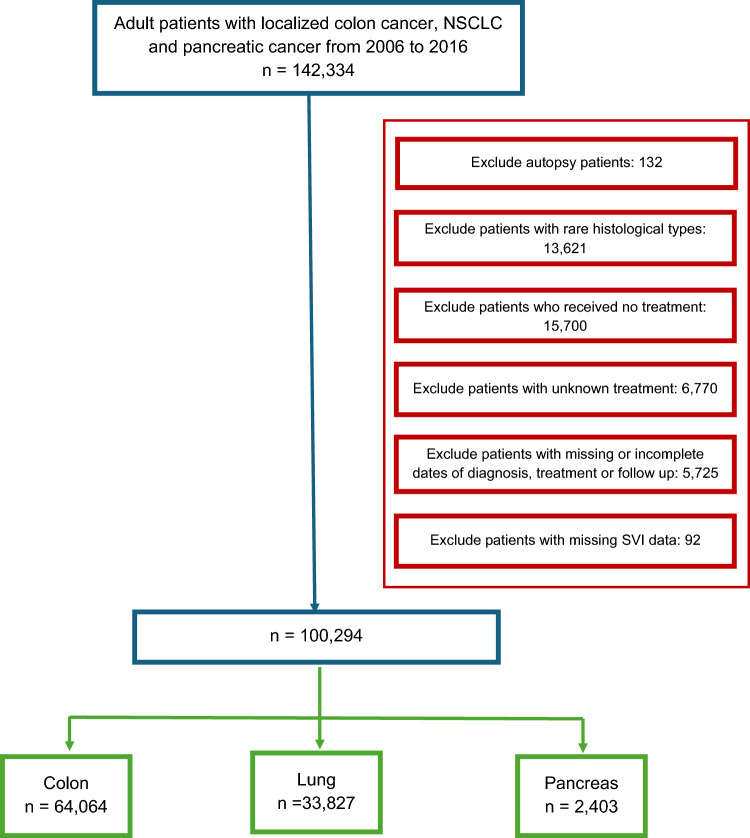
Flow diagram of patients included after application of inclusion and exclusion criteria. NSCLC, non-small-cell lung cancer; SVI, Social Vulnerability Index

Patients aged ≥18 years and diagnosed with colon cancer, NSCLC, or pancreatic cancer were identified using International Classification of Diseases for Oncology, third edition (ICD-O-3) topography codes (Fig. 1). We used the Surveillance Epidemiology and End Results (SEER) summary staging system to identify and include patients who were staged as “localized”. This avoided selection bias and allowed us to include patients who had the most limited disease, required the least complicated care, and had the longest survival (details in the supplementary material). Since our main predictors were treatment characteristics and SVI, we excluded patients who did not get any cancer treatment (11.0%) or were missing SVI information (0.06%) (Fig. 1).

### Variable Selection

**Fig. 2:**
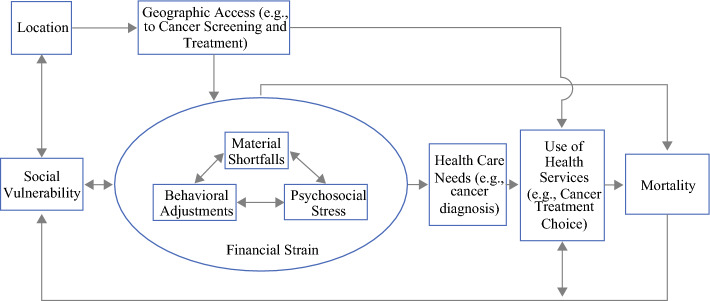
Conceptual model: Andersen’s Behavioral Model of Health Services Use and models of financial strain experienced by patients with cancer

We expanded Andersen’s Behavioral Model of Health Services Use ^11^ and models of financial strain experienced by patients with cancer^12^ to elucidate the role of economic and geographic factors in patients’ cancer treatment choices (Fig. 2). Where patients live affects both their geographic access to cancer care and their exposure to social vulnerability. Both affect the way in which patients experience financial strain, which then affects how they use health services. The model illustrates how the use of health services is on the pathway linking SVI and mortality.

### Exposures of Interest

*Cancer Care Delivery* Two attributes of cancer care delivery were included in this variable. The guideline concordance of treatment modalities was defined using multiple clinical management guidelines published during the study period (National Comprehensive Care Network, the American Society of Colon and Rectal Surgeons, American College of Chest Physicians, Society of Thoracic surgeons, American Society of Clinical Oncology, and others) as follows: surgical resection excluding endoscopic removal or diversion-only operations for colon cancer; surgical resection or radiation for NSCLC; and surgical resection with chemotherapy for pancreatic cancer.^13–15^ Treatment initiation was defined as either timely or delayed if it started within or after 60 days from the date of diagnosis, respectively, and was calculated as the difference between receipt of first treatment and date of diagnosis. This was based on prior research, where initiation after 60 days was associated with worse survival, and as a reflection of practice patterns, where further delay can be considered unreasonable.^4^ The two attributes were combined into four categories: one optimal care category—treatment that was both guideline concordant and timely; three non-optimal care categories (i: treatment that was timely but not guideline concordant; ii: treatment that was guideline concordant but not timely; and iii: treatment that was neither timely nor guideline concordant).

*SVI* SVI was analyzed as both a continuous and a categorical variable. As a continuous variable, SVI was scaled so that it ranged from 0 to 10; the SVI measure divided by 10 corresponds to a census tracts percentile ranking (i.e., SVI=1 specifies a census tract at the 10th percentile). The categorical variable was formed using percentile ranks to create tertiles. Tertiles were used as they facilitated the categorization of interaction variables and were consistent with recently published studies that investigated SVI and cancer mortality.^16^ Patients were characterized as residing in the least vulnerable (lowest SVI tertile), less vulnerable (mid SVI tertile), or most vulnerable (highest SVI tertile) neighborhoods. These were first analyzed as tertile categories and then used as a binary variable for the interaction analysis where we compared outcomes for patients who resided in the most vulnerable neighborhoods (highest SVI tertile) with those who lived in less/least vulnerable neighborhoods (mid/lowest SVI tertile). This is further described below.

*Composite Categorical Exposure* To examine how the association of optimal care with survival varied in neighborhoods with different levels of social vulnerability, we first used two categories each to characterize treatment (optimal care or non-optimal care) and neighborhoods (most vulnerable neighborhoods [highest SVI tertile] vs less/least vulnerable neighborhoods [mid and lowest SVI tertile]). Then, we created four categories to characterize patient’s joint situations: optimal care and most vulnerable neighborhoods, optimal care and less/least vulnerable neighborhoods, non-optimal care and most vulnerable neighborhoods, and non-optimal care and most vulnerable neighborhoods.

### Covariates

The following additional covariates were included in all models: age in years, gender (male, female, or non-binary), the US State (Texas or California), race and ethnicity (non-Hispanic white, non-Hispanic Black, non-Hispanic Asian, non-Hispanic of other races, Hispanic, or unknown race/ethnicity), insurance status (uninsured, privately insured, Medicare, Medicaid, other Governmental insurance plans, insurance not otherwise specified, and unknown), and period of diagnosis (2006–2009, 2010–2013, 2014–2016). The race and ethnicity variables were used to control for the disadvantages that have resulted from structural and systemic discrimination faced by racial minorities as these may coexist with neighborhood vulnerabilities. Missing data for covariates were small in number (<1.5%) and reported as separate categories.

### Outcome

The primary outcome of interest was all-cause mortality and overall survival 5 years from diagnosis.

### Statistical Analyses

All analyses were stratified by cancer type. Time-to-death analysis comparisons among patients receiving different levels of cancer care were conducted by calculating survival rates and plotting them using Kaplan–Meier curves. Log-rank tests were used to test for statistical significance of differences in survival. A multivariable Cox proportional hazards regression model was used to determine the association between cancer care delivery, SVI, and control variables with all-cause mortality. In our models, we controlled for patient age, insurance, sex, and race/ethnicity to limit the confounding of these patient-level characteristics known to be associated with mortality. To determine the interplay between cancer care delivery and SVI, the combined composite categorical variable was used in the Cox proportional hazard model, fully adjusted for control variables. Kaplan–Meier survival curves were developed to describe the relationship between the composite categorical variable and survival for each cancer. StataCorp (2023; Stata Statistical Software: Release 18; College Station, TX: StataCorp LLC) was used for quantitative analysis with *p*-values <0.05 considered significant.

## Results

### Descriptive Analysis

The study included 100,294 patients: 63,661 (63.5%) from California and 36,633 (36.5%) from Texas (Table 1). Colon cancer was the most common (64,064 [63.8%]) and pancreatic cancer the least common (2591 [2.4%]). In total, 69% of patients were non-Hispanic white. The mean age of diagnosis was >65 years for all cancer types. This was reflected in the insurance status as Medicare was the most common insurance type, and only 2.2% were uninsured. Patients with NSCLC were least likely to receive timely care (72.8%) but most likely to receive guideline-concordant care (96.6%). Of these patients, 68% underwent surgery, 26% received chemotherapy, and 1.9% received both. Patients with pancreatic cancer were least likely to receive guideline-concordant care (24.9%) as most patients just received chemotherapy (46.8%) or surgery (28.2%). After combining the two cancer care delivery attributes, patients with colon cancer were most likely (83.7%) and patients with pancreatic cancer (22.6%) least likely to receive optimal care. Similarly, when assessing patients receiving optimal care based on social vulnerability, we observed the following rates for each cancer when comparing the least vulnerable to the most vulnerable: colon cancer 85.2% versus 81.4%, *p *< 0.001; NSCLC 74.6% versus 65.3%, *p *< 0.001; pancreatic cancer 25.2% versus 17.3%, *p *= 0.002.

**Table 1:** Patient and cancer care characteristics by cancer type

Variables		Overall	Colon	Lungs	Pancreas
		(*n* = 100,294)	(*n* = 64,064)	(*n* = 33,827)	(*n* = 2403)
Age		69.1 ± 12.0	68.2 ± 12.9	70.8 ± 9.7	67.8 ± 13.1
*Sex*					
Male		49,596 (49.4)	32,569 (50.8)	15,923 (47.1)	1119 (46.6)
Female		50,683 (50.5)	31,495 (49.2)	17,804 (52.9)	1284 (53.4)
*State of diagnosis*					
California		63,661 (63.5)	40,482 (63.2)	21,638 (63.9)	1541 (64.1)
Texas		36,633 (36.5)	23,582 (36.8)	12,189 (36.0)	862 (35.9)
*Race/Hispanic ethnicity*					
Non-Hispanic white		68,956 (68.7)	41,139 (64.2)	26,291 (77.7)	1526 (63.5)
Non-Hispanic Black		8483 (8.5)	5841 (9.1)	2433 (7.2)	209 (8.7)
Non-Hispanic Asian		6793 (6.8)	4531 (7.1)	1066 (6.1)	196 (8.2)
Non-Hispanic of other races		1746 (1.7)	1219 (1.9)	470 (1.4)	57 (2.4)
Hispanic		13,925 (13.9)	10,970 (17.1)	2541 (7.5)	415 (17.2)
Unknown race/ethnicity		391 (0.4)	364 (0.6)	26 (0.1)	-
*Insurance status*					
Private		34,786 (34.7)	24,236 (37.8)	9,695 (28.7)	855 (36.6)
Uninsured		2211 (2.2)	1736 (2.7)	407 (1.2)	68 (2.8)
Medicaid		4510 (4.5)	3,181 (4.9)	1216 (3.6)	113 (4.7)
Medicare		49,607 (49.5)	28,992 (45.2)	19,489 (57.6)	1126 (46.9)
VA/Tricare/military/IHS		1310 (1.3)	624 (0.9)	646 (1.9)	40 (1.7)
Insurance NOS		3567 (3.6)	2488 (3.9)	976 (2.9)	103 (2.3)
Unknown/missing		4303 (1.3)	2807 (4.4)	1398 (4.1)	98 (4.1)
*Cancer care delivery*					
Delayed treatment initiation		12,887 (12.8)	3310 (5.2)	9,192 (27.2)	385 (16.0)
Guideline-concordant care		90,069 (89.8)	56,780 (88.6)	32,690 (96.6)	599 (24.9)
*Combined treatment initiation and guideline concordance*					
Non-optimal care	Delayed and not GCC	835 (0.8)	125 (0.2)	381 (1.1)	329 (13.7)
	Timely and not GCC	9390 (9.4)	7159 (11.2)	756 (2.2)	1475 (61.4)
	Delayed and GCC	12,052 (12.0)	3185 (4.9)	8811 (26.0)	56 (2.3)
Timely and GCC (optimal care)		78,017 (77.8)	53,595 (83.7)	23,879 (70.6)	543 (22.6)

*GCC* guideline-concordant care, *IHS* Indian health service, *NOS* not otherwise specified, *VA* veterans’ affairs

Patients who received optimal care had the highest rates of survival at 5 years for all cancer types when compared with non-optimal categories (Table 2). The 5-year survival rates for optimal care when compared with the least optimal care (non-guideline concordant and not timely) were as follows: colon cancer 76.4% versus 50.1%, *p *< 0.001; NSCLC 57.2% versus 23.9%, *p *< 0.001; pancreatic cancer 46.2% versus 13.4%, *p *< 0.001.

**Table 2:** 5-year survival for each treatment category, stratified by cancer type

Cancer types	Overall	Non-optimal care			Optimal care: timely and GCC
		Delayed and non-GCC (least optimal)	Timely and non-GCC	Delayed and GCC	
Colon	75.9	50.1	74.4	71.8	76.4
Lung	53.3	23.9	17.2	46.8	57.2
Pancreas	27.7	13.4	23.9	28.4	46.2

Data are presented as percentages. Difference in survival is significantly different between the treatment categories (*p *< 0.001)

*GCC* guideline-concordant care

### Multivariable Survival Analysis

Multivariable Cox proportional hazard models showed that each additional year of age at diagnosis increased the hazard of death by 3–7% (Table 3). The estimated hazard of death was 12–27% lower for female patients than for male patients and 19–35% lower for non-Hispanic Asian patients than for non-Hispanic white patients. Compared with private insurance, all insurance types were significantly associated with higher mortality for patients with colon cancer and NSCLC. Patients with Medicaid insurance had a 25–69% greater mortality risk than privately insured patients. Similarly, uninsured patients had a 24–52% higher risk of death than privately insured patients. No insurance type was associated with a statistically significant difference in survival for patients with pancreatic cancer.

**Table 3:** Multivariable Cox proportional hazards for all-cause mortality, stratified by cancer type

Characteristic	Variable	Colon	Lung	Pancreas
Age	1.07 (1.07–1.07)	1.04 (1.04–1.04)	1.03 (1.03–1.04)	
Sex	Male	Ref	Ref	Ref
	Female	0.76 (0.75–0.78)	0.73 (0.71–0.75)	0.88 (0.81–0.97)
US state of	California	Ref	Ref	Ref
diagnosis	Texas	1.002 (0.97–1.03)	1.04 (1.01–1.07)	1.15 (1.04–1.27)
Race/hispanic ethnicity				
	Non-Hispanic white	Ref	Ref	Ref
	Non-Hispanic Black	1.07 (1.02–1.12)	0.98 (0.93–1.04)	1.06 (0.89–1.27)
	Non-Hispanic Asian	0.77 (0.73–0.82)	0.65 (0.61–0.70)	0.81 (0.67–0.97)
	Non-Hispanic/other races	0.60 (0.53–0.68)	0.66 (0.57–0.77)	0.66 (0.47–0.93)
	Hispanic	0.91 (0.88–0.95)	0.87 (0.82–0.92)	0.95 (0.83–1.09)
	Unknown race/ethnicity	0.09 (0.05–0.18)	1.06 (0.98–1.14)	-
Insurance status	Private	Ref	Ref	Ref
	Uninsured	1.51 (1.37–1.67)	1.24 (1.08–1.43)	0.88 (0.63–1.23)
	Medicaid	1.68 (1.56–1.79)	1.39 (1.27–1.50)	1.25 (0.96–1.62)
	Medicare	1.23 (1.19–1.27)	1.10 (1.06–1.14)	1.00 (0.89–1.12)
	VA/Tricare/Military	1.30 (1.13–1.50)	1.21 (1.09–1.34)	0.82 (0.56–1.19)
	Insurance NOS	1.07 (0.98–1.15)	1.06 (0.97–1.16)	0.73 (0.56–0.96)
	Unknown/missing	1.25 (1.18–1.34)	1.06 (0.98–1.14)	1.04 (0.82–1.32)
Diagnosis periods	2006–2009	Ref	Ref	Ref
	2010–2013	1.02 (0.99–1.05)	0.94 (0.91–0.97)	0.95 (0.85–1.06)
	2014–2016	0.98 (0.94–1.02)	0.84 (0.81–0.87)	0.89 (0.79–1.01)
SVI	1.05 (1.04–1.05)	1.04 (1.04–1.05)	1.02 (0.99–1.-03)	
Cancer care delivery	Delayed and not GCC	Ref	Ref	Ref
	(least optimal care)			
	Timely and not GCC	0.57 (0.45–0.72)	1.29 (1.13–1.48)	0.88 (0.77–1.005)
	Delayed and GCC	0.51 (0.40–0.65)	0.55 (0.49–0.61)	0.61 (0.44–0.84)
	Timely and GCC (optimal care)	0.45 (0.37–0.57)	0.43 (0.39–0.48)	0.46 (0.39–0.54)

*GCC* guideline-concordant care, *NOS* not otherwise specified, *SVI* Social Vulnerability Index, *VA* veterans’ affairs

From the multivariable models that used SVI as a continuous variable, for each 10-unit increase in SVI, the hazard of death increased by 5% for colon cancer (*p *< 0.001), 4% for NSCLC (*p *< 0.001), and 2% for pancreatic cancer (*p *= 0.06) (Table 3). From the multivariable models that used SVI as a categorical variable, for patients from the most vulnerable neighborhoods compared with those living in the least vulnerable neighborhoods, the hazard of death was 30% higher for NSCLC and 33% higher for colon cancer (Supplementary Table 1). For pancreatic cancer, the hazard of death was also higher for patients in less vulnerable (17%, *p *= 0.003) and most vulnerable (12%, *p *= 0.07) neighborhoods when compared with the least vulnerable neighborhoods.

### Interaction of Cancer Care Delivery with SVI

**Fig. 3:**
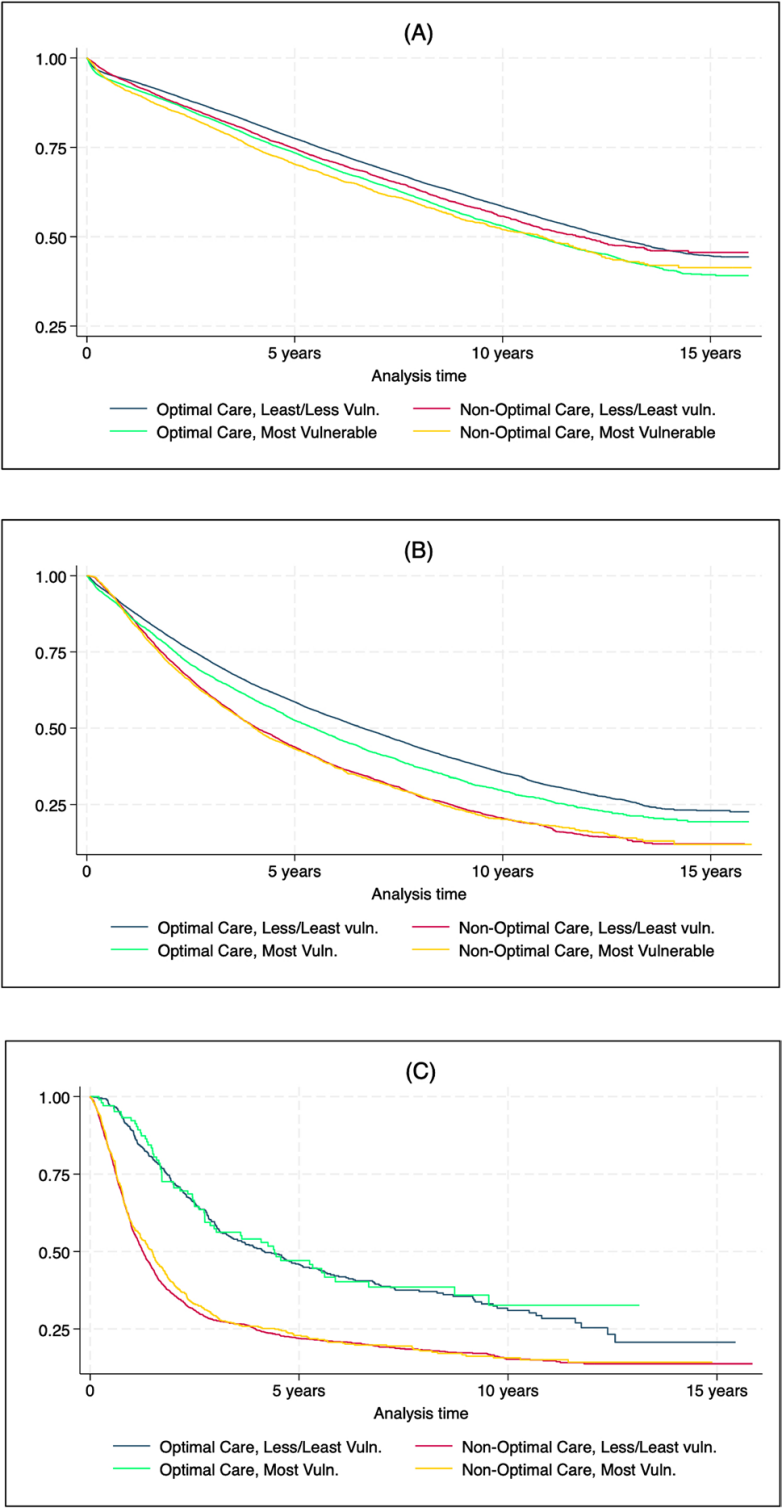
Kaplan–Meier survival estimates by care type and Social Vulnerability Index (SVI) for **A** colon cancer, **B** lung cancer, and **C** pancreatic cancer

Results from this analysis are presented in Table 4. The Kaplan–Meier survival curve (Fig. 3) illustrates the combined relationship of optimal care and SVI with cancer survival. For NSCLC and pancreatic cancer, patients who received non-optimal care had the worst survival, and rates were similar for patients from the most vulnerable and less/least vulnerable neighborhoods. For NSCLC and pancreatic cancer, the hazard of death with receipt of non-optimal care increased from 43% (non-optimal care, less/least vulnerable neighborhoods for NSCLC) to 98% (non-optimal care, most vulnerable neighborhood for pancreatic cancer). For pancreatic cancer, survival rates for patients receiving optimal care were also similar across neighborhood types. However, among those receiving optimal care for colon cancer and NSCLC, the hazard of death was 23% and 24% higher, respectively, for patients living in the most vulnerable neighborhoods than for those in the less/least vulnerable neighborhoods. Overall, although survival with non-optimal care was similar across neighborhood types, patients from the most vulnerable neighborhoods did not gain maximum survival benefits from optimal care.

**Table 4:** Multivariable Cox proportional hazards model^a^ for all-cause mortality, stratified by cancer type (with composite categorical variable that combines cancer care delivery and Social Vulnerability Index [SVI])

Variable	Colon	Lungs	Pancreas
Cancer care delivery and SVI			
Optimal care, less/least vulnerable neighborhoods	Ref	Ref	Ref
Non-optimal care, less/least vulnerable neighborhoods	1.25 (1.20–1.30)	1.43 (1.38–1.48)	1.90 (1.66–2.17)
Optimal care, most vulnerable neighborhoods	1.23 (1.19–1.27)	1.24 (1.19–1.29)	0.98 (0.74–1.30)
Non-optimal care, most vulnerable neighborhoods	1.44 (1.36–1.53)	1.57 (1.49–1.65)	1.98 (1.68–2.33)

^a^Model adjusted for age, gender, race/ethnicity, insurance status, diagnosis period, and US state at diagnosis

## Discussion

In this study, receiving optimal cancer care (timely and guideline concordant) and neighborhood vulnerability were both independently associated with cancer survival. The greatest survival benefit was observed in patients who received both timely and guideline-concordant care and resided in the less and least vulnerable neighborhoods. Thus, our results suggest that advantageous combinations of both upstream and downstream factors are important for optimal cancer survival. The Kaplan–Meier curves in Fig. 2 highlight the primary importance of receiving optimal cancer care, especially for NSCLC and pancreatic cancer. However, current cancer care guidelines lack specific recommendations on time to treatment initiation, potentially excluding it from treatment planning and leading to prolonged delays. This is an important consideration for socially vulnerable patients who face barriers to cancer care.^17^

Residence in a socially vulnerable neighborhood was independently associated with worse survival. Even when patients from these neighborhoods received optimal care, their survival was lower than for patients who lived in the less/least vulnerable neighborhoods. The underlying mechanisms of these relationships involve both upstream and downstream factors. Upstream socio-political power and values create systems that may affect access to resources and opportunities.^2^ These are reinforced by current or historic federal, state, county, and city laws, regulations, and policies that may disadvantage residents of socially vulnerable neighborhoods long after the legal statutes have changed.^18^ This creates barriers, many of which are part of SVI and have also been identified by other studies. These include transportation, housing insecurity and crowding, lower education level, disabilities, poverty, unemployment, and language barriers.^19,20^ These factors can compromise the quality of cancer and survivorship care and result in the development or worsening of other medical conditions, leading to shortened overall survival.

Our results indicate that, to deliver effective cancer care equitably, we will likely have to address both downstream and upstream factors. The healthcare system and facilities may consider steps to mitigate at least some of these disparities. These can include improving care coordination and navigation between hospitals and specialists, expediting staging work-up, facilitating transportation, and addressing language barriers, among others. State health officials can leverage state cancer registry data to monitor and report quality metrics, including time to treatment initiation and adherence to clinical guidelines. As demonstrated in our study, both factors when optimal together result in the longest survival in patients with cancer. This aligns with existing literature, which shows that even a single week of treatment delay is associated with an increased absolute risk of mortality.^6,21^ Addressing factors that create social vulnerability, such as education, income, employment, and nutrition, through upstream policy changes may result in overall health improvement and allow patients to realize the full benefit of optimal care. Indeed, it is important that the findings from this study are interpreted within the context of intersectionality, that is, that even within the same neighborhood, multiple overlapping social factors may create disparities in individuals’ access to resources, possibly influencing long-term outcomes. Positioning our findings within the National Cancer Plan makes them more policy relevant as they highlight how intersecting social vulnerabilities are related with cancer treatment and survival. By aligning our results with established priorities, we underscore that accounting for community-level vulnerabilities may enhance policy effectiveness and potentially improve survival.

Our findings align with other research on the relationship between SVI and cancer care and outcomes. A recent nationwide study using the Centers for Disease Control and Prevention’s SVI and Wide-ranging Online Data for Epidemiologic Research database found a significant association between SVI and age-adjusted mortality rates for multiple cancers.^22^ Meier et al. found that compared to lower SVI, a higher SVI was associated with lower rates of surgical resection and increased mortality for patients with localized colon cancer.^23^ Similarly, Chan et al. reported that compared to low SVI counties, colorectal cancer patients from high SVI counties were less likely to receive  guideline concordant care.^24^ Stuart et al. found that compared to NSLC patients from less vulnerable neighborhoods, those from more vulnerable neighborhoods presented with advanced disease and had lower odds of undergoing surgery.^25^ Azap et al. found that older Medicare beneficiaries in high SVI counties were less likely to have surgery when compared to beneficiaries from low SVI counties.^26^ However, most of these studies are limited by the use of county-level SVI data. This study uses census-tract-level SVI, which better reflects patients’ neighborhood characteristics. Furthermore, although patients with pancreatic cancer in our study did not show an association with SVI, this observation may reflect broader trends seen in the literature. Pancreatic cancer is known for its asymptomatic nature, which results in 53–60% of patients presenting with metastatic disease, which has inherently poor outcomes. Additionally, pancreatic cancer has historically been subjected to a nihilistic approach with very low treatment rates in recently reported literature.^27–29^ The challenges in pancreatic cancer treatment that stem from these factors and their interaction with social vulnerability warrant further investigation.

This study is meaningful, but it should be viewed with the limitations that apply. Consistent with the study’s objective, we excluded patients who did not receive treatment. Bivariate analysis shows that the proportion of patients who did not receive treatment increased significantly as neighborhood vulnerability increased (Supplementary Table 2). So, although the findings are valid for this study’s objective, they do not fully estimate the relationship of neighborhood vulnerability with other aspects of cancer care. A separate study that investigates the factors associated with no cancer treatment will be able to further explore this relationship. Furthermore, in our models, we controlled for patient insurance status to account for some aspects of financial strain, although we acknowledge as a limitation that more detailed data preclude complete control of this factor so results could reflect differences in financial strain experienced by patients living in higher versus lower SVI neighborhoods. The methodology identified strong associations but cannot infer causality. Moreover, including patients diagnosed until 2016 allowed a longer survival follow-up but raises the question about changes in relationship between social vulnerability and cancer treatment or survival that may have since occurred. Population-based cancer databases are limited by the granularity of data they contain. For this reason, the outcome studied is all-cause mortality and not cancer-specific mortality. This approach better captures the impact of social factors since vulnerable cancer survivors may face non-cancer-related health issues affecting overall survival. For the same reason, we could not include factors such as medical comorbidities, access to cancer care, changes in insurance status after treatment initiation, treatment quality, reasons for treatment delays, or delays in treatment with certain modalities. This level of granularity requires prospectively collected or hospital-level data that offer local insights but lack generalizability. The use of population-based data allows this study to estimate average association at the population level over a prolonged period.

Patients with localized colon cancer, NSCLC, or pancreatic cancer who receive both timely and guideline-concordant care survive the longest. However, this survival benefit is lower for patients who reside in vulnerable neighborhoods, indicating an association between social factors and patients’ overall health and care after being diagnosed with cancer. Although efforts should focus on delivering optimal care to all patients, addressing the social factors in vulnerable neighborhoods is essential to improve overall survival.

## Supplementary Information

Below is the link to the electronic supplementary material.

Supplementary file1 (DOCX 25 KB)
