# Supplementary material for: Combined Association of Time to Treatment, Guideline Concordance, and Neighborhood Vulnerability with Localized Colon Cancer, Non-small-cell Lung Cancer, and Pancreatic Cancer Survival
Source: Ann Surg Oncol. 2026 May 17;33(8):7386–95. doi: 10.1245/s10434-026-19760-5 (PMC13337619; doi:10.1245/s10434-026-19760-5)
Supplement: Supplementary file 1 — Supplementary file1 (DOCX 25 KB) [file 10434_2026_19760_MOESM1_ESM.docx]

Supplementary Methods:

Considerations for including localized cancer patients:

**Why did we only include localized cancers?**

- Localized cancer patients have the best overall survival while requiring, in some cases, relatively less complicated staging workup and less cancer-directed therapy. They are also less likely to face delays that result from seeking specialized cancer care, like multi-specialty tumor board discussions, that are often necessary for cancers that have regional involvement.
- Arguably, these factors may make patients with localized cancer relatively less sensitive to social barriers in acquiring optimal care, thus making them ideal for this study. Even with the most limited disease that requires the least complicated cancer care, we show that survival is different in cases where patients don’t receive optimal care and come from vulnerable neighborhoods.

**Why did we not include regional cancers that involved Lymph nodes?**

- SEER Summary Stage uses all information available in the first four months of diagnosis, which also includes operative findings. Since the nodal staging is most accurately done from an operation, the likelihood of patients to be categorized as having regional nodal involvement is higher for patients who undergo surgery. Including these patients would have biased our analytic sample to those who received surgical resection negatively impacting the results.

**Why did we not include regional cancers that involved adjacent structures?**

- SEER staging does not differentiate between the involvement of structures that are surgically resectable from structures that makes the cancer unresectable. For example, ampulla of Vater involvement in pancreatic cancer is grouped together with pancreatic cancers that involve major blood vessels

To summarize, by including localized cancer, we select patients who have the most limited disease, require the least complicated cancer care, and have the longest survival while also allowing us to maintain consistency across the cancer types in terms of staging and guideline concordance definitions.

Supplementary Table 1: Multivariable Cox Proportional Hazards for all-cause mortality stratified by cancer type with SVI as categorical variable.

|  | Variables | Colon | Lung | Pancreas |
| --- | --- | --- | --- | --- |
| Age | | 1.07 (1.07 – 1.07) | 1.04 (1.04 – 1.04) | 1.03 (1.03 – 1.04) |
| Gender | Male  Female | Ref  0.76 (0.75 – 0.78) | Ref  0.73 (0.71 – 0.75) | Ref  0.88 (0.81 – 0.97) |
| US State of Diagnosis | California  Texas | Ref  1.005 (0.98 – 1.03) | Ref  1.04 (1.01 – 1.07) | Ref  1.15 (1.04 – 1.27) |
| Race/Hispanic Ethnicity | Non-Hispanic White  Non-Hispanic Black  Non-Hispanic Asian  Non-Hispanic/Other Races  Hispanic  Unknown Race/Ethnicity | Ref  1.09 (1.04 – 1.14)  0.79 (0.73 – 0.82)  0.60 (0.53 – 0.69)  0.93 (0.89 – 0.97)  0.09 (0.05 – 0.18) | Ref  0.98 (0.93 – 1.04)  0.65 (0.61 – 0.70)  0.66 (0.57 – 0.77)  0.87 (0.82 – 0.92)  1.06 (0.98 – 1.14) | Ref  1.06 (0.89 – 1.27)  0.81 (0.67 – 0.97)  0.66 (0.47 – 0.93)  0.95 (0.83 – 1.09)  - |
| Insurance Status | Private  Uninsured  Medicaid  Medicare  VA/Tricare/Military  Insurance NOS  Unknown/Missing | Ref  1.52 (1.37 – 1.67)  1.69 (1.58 – 1.82)  1.23 (1.19 – 1.27)  1.30 (1.13 – 1.50)  1.07 (0.99 – 1.16)  1.25 (1.17 – 1.34) | Ref  1.24 (1.08 – 1.43)  1.39 (1.27 – 1.50)  1.10 (1.06 – 1.14)  1.21 (1.09 – 1.34)  1.06 (0.97 – 1.16)  1.06 (0.98 – 1.14) | Ref  0.88 (0.63 – 1.23)  1.25 (0.96 – 1.62)  1.00 (0.89 – 1.12)  0.82 (0.56 – 1.19)  0.73 (0.56 – 0.96)  1.04 (0.82 – 1.32) |
| Diagnosis Periods | 2006 – 2009  2010 – 2013  2014 – 2016 | Ref  1.02 (0.99 – 1.05)  0.98 (0.94 – 1.02) | Ref  0.94 (0.91 – 0.97)  0.84 (0.81 – 0.87) | Ref  0.95 (0.85 – 1.06)  0.89 (0.79 – 1.01) |
| SVI | Least Vulnerable  Less Vulnerable  Most Vulnerable | Ref  1.17 (1.13 – 1.21)  1.33 (1.29 – 1.38) | Ref  1.17 (1.13 – 1.21)  1.30 (1.25 – 1.35) | Ref  1.17 (1.06 – 1.31)  1.12 (0.99 – 1.28) |
| Cancer Care Delivery | Delayed and not GCC  (Least Optimal Care)  Timely and not GCC  Delayed and GCC  Timely and GCC (Optimal care) | Ref  0.57 (0.45 – 0.72)  0.51 (0.40 – 0.65)  0.45 (0.37 – 0.57) | Ref  1.29 (1.13 – 1.48)  0.55 (0.49 – 0.61)  0.43 (0.39 – 0.48) | Ref  0.88 (0.77 – 1.005)  0.61 (0.44 – 0.84)  0.46 (0.39 – 0.54) |

Supplementary Table 2: Distribution of neighborhood SVI by receipt of treatment, overall and stratified by cancer type.

| Cancer Types | Overall* | | | Colon* | | | Lung* | | | Pancreas* | | |
| --- | --- | --- | --- | --- | --- | --- | --- | --- | --- | --- | --- | --- |
|  | Received Treatment  (Analytic Sample) | No Treatment | Unknown Treatment Status | Received Treatment  (Analytic Sample) | No Treatment | Unknown Treatment Status | Received Treatment  (Analytic Sample)t | No Treatment | Unknown Treatment Status | Received Treatment  (Analytic Sample) | No Treatment | Unknown Treatment Status |
|  | 100,294 | 15,700 | 12,587 | 64,064 | 3,370 | 4,145 | 33,827 | 8,878 | 7,685 | 2,403 | 3,452 | 757 |
| Least Vulnerable | 35,896  (80.5) | 4,710  (10.6) | 3,965  (8.9) | 22,244  (90.3) | 963  (3.9) | 1,430  (5.8) | 12,721  (72.1) | 2,648  (15.0) | 2,276  (12.9) | 931  (40.7) | 1,099  (48.0) | 259  (11.3) |
| Less Vulnerable | 37,540  (78.0) | 5,838  (12.1) | 4,740  (9.8) | 23,710  (89.8) | 1,228  (4.6) | 1,473  (5.6) | 12,969  (67.1) | 3,344  (17.3) | 3,003  (15.5) | 861  (36.0) | 1,266  (52.9) | 264  (11.0) |
| Most Vulnerable | 26,858  (74.8) | 5,152  (14.3) | 3,882  (10.8) | 18,110  (88.2) | 1,179  (5.7) | 1,242  (6.0) | 8,137  (60.6) | 2,886  (21.5) | 2,406  (17.9) | 611  (31.6) | 1,087  (56.3) | 234  (12.1) |

*Differences across the SVI Neighborhood categories are statistically significant with p<0.001
